# Atrial septal defect with a rare occupying lesion in heart

**DOI:** 10.1186/s12872-022-02919-9

**Published:** 2022-11-12

**Authors:** Jinlan Chen, Xueyang Gong, Li Xie, Qin Wu, Tianli Zhao, Shijun Hu

**Affiliations:** Department of Cardiovascular Surgery, the Second Xiangya Hospital, Central South University, Changsha, 410011 P. R. China

**Keywords:** Cardiac tumors, Cardiac epicardium hemangiomas, Congenital heart disease, Biopsy, Infant

## Abstract

**Background:**

Cardiac epicardium hemangiomas are exceedingly rare; however, they can cause significant hemodynamic impairment and large pericardial effusions. On rare occasion, cardiac tumors coexist with malformations of the heart.

**Case presentation:**

We present the case of a 10-month-old female infant with a rare cardiac surface hemangioma coexisting with malformations of the heart. It revealed an atrial septal defect (ASD) coexisting with an abnormal occupying lesion with high echogenicity. It was 35*12*9 mm in size and was found in the anterior atrioventricular junction to the posterior atrioventricular junction at the bottom of the ventricular septum by transthoracic echocardiography. We performed surgical treatment of the atrial septal defect and performed biopsy with the occupying lesion. The histopathological examination reported a benign tumor as hemangioma. As far as we know, this is the first case in which cardiac surface hemangioma was found to coexist with an atrial septal defect.

**Conclusions:**

Cardiac epicardium hemangiomas is a rare solid tumor of the heart. If the mass is impossible to resect and does not cause hemodynamic impairment, only mass biopsy is possible.

## Background

Primary cardiac tumors are exceedingly rare in the pediatric population, with an estimated prevalence ranging from 0.027 to 0.49%, and cardiac hemangiomas are even rarer. Although cardiac hemangiomas are histologically benign and may even regress spontaneously, they can cause significant complications including large pericardial effusions, congestive heart failure, inflow or outflow tract obstructions, and disturbances in heart rhythm in some cases. Rarely, cardiac hemangiomas may also coexist with congenital heart lesions, in particular cardiac epicardium hemangiomas. Herein, we report a very rare case of a cardiac epicardium hemangiomas with atrial septal defect in a 10-month-old female infant.

## Case presentation

**Fig. 1:**
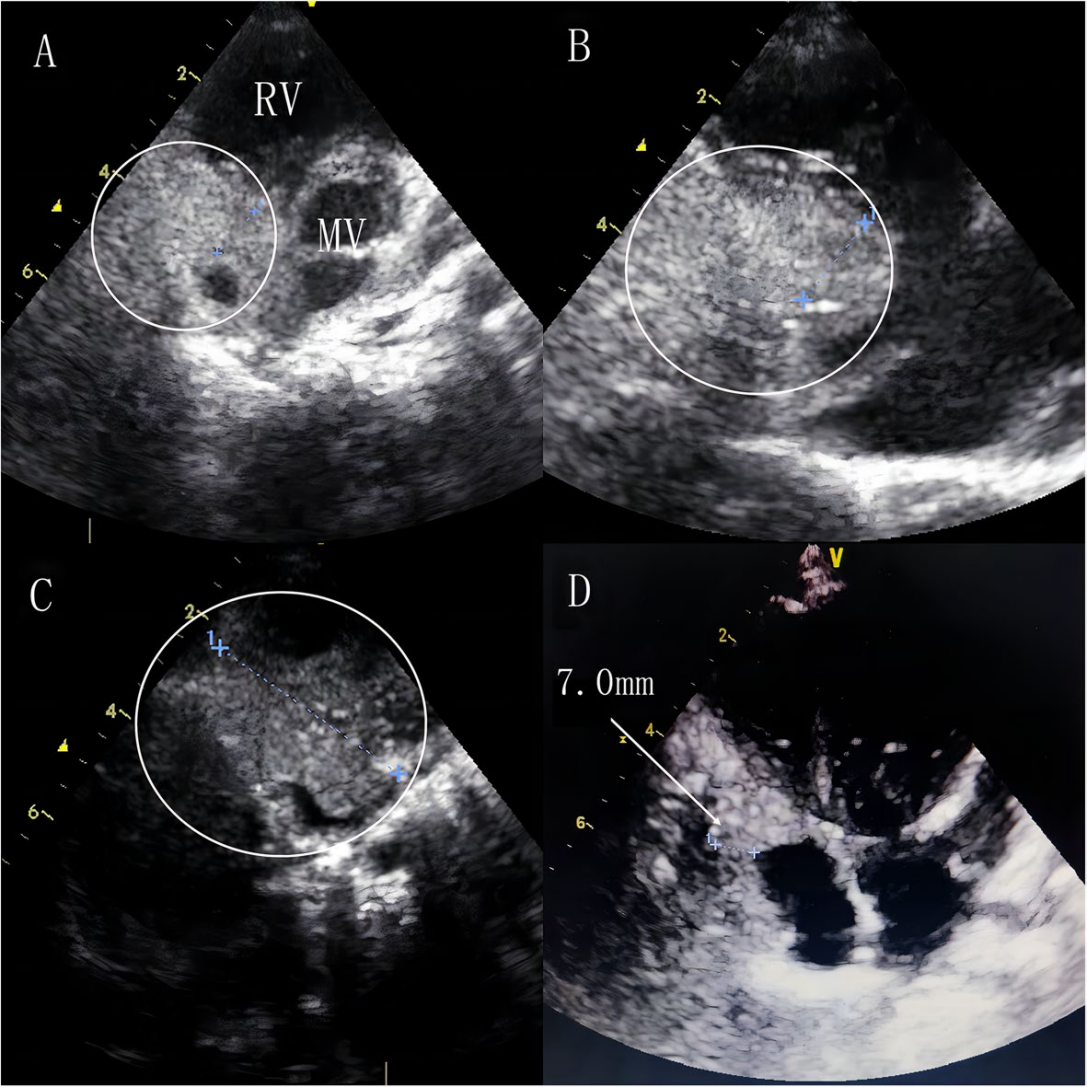
**A** Transthoracic echocardiogram obtained in the parasternal non-standard four-chamber view showing the mass with uneven echo. **B** Transthoracic echocardiogram obtained in the right ventricular inflow tract view showing the mass and the atrial septal defect (white arrow). **C** Transthoracic echocardiogram obtained in the Subxiphoid view showing the mass. **D, E** Transthoracic echocardiogram showing the mass that has developed two years after the surgery. MV, mitral valve; RV, right ventricle

**Fig. 2:**
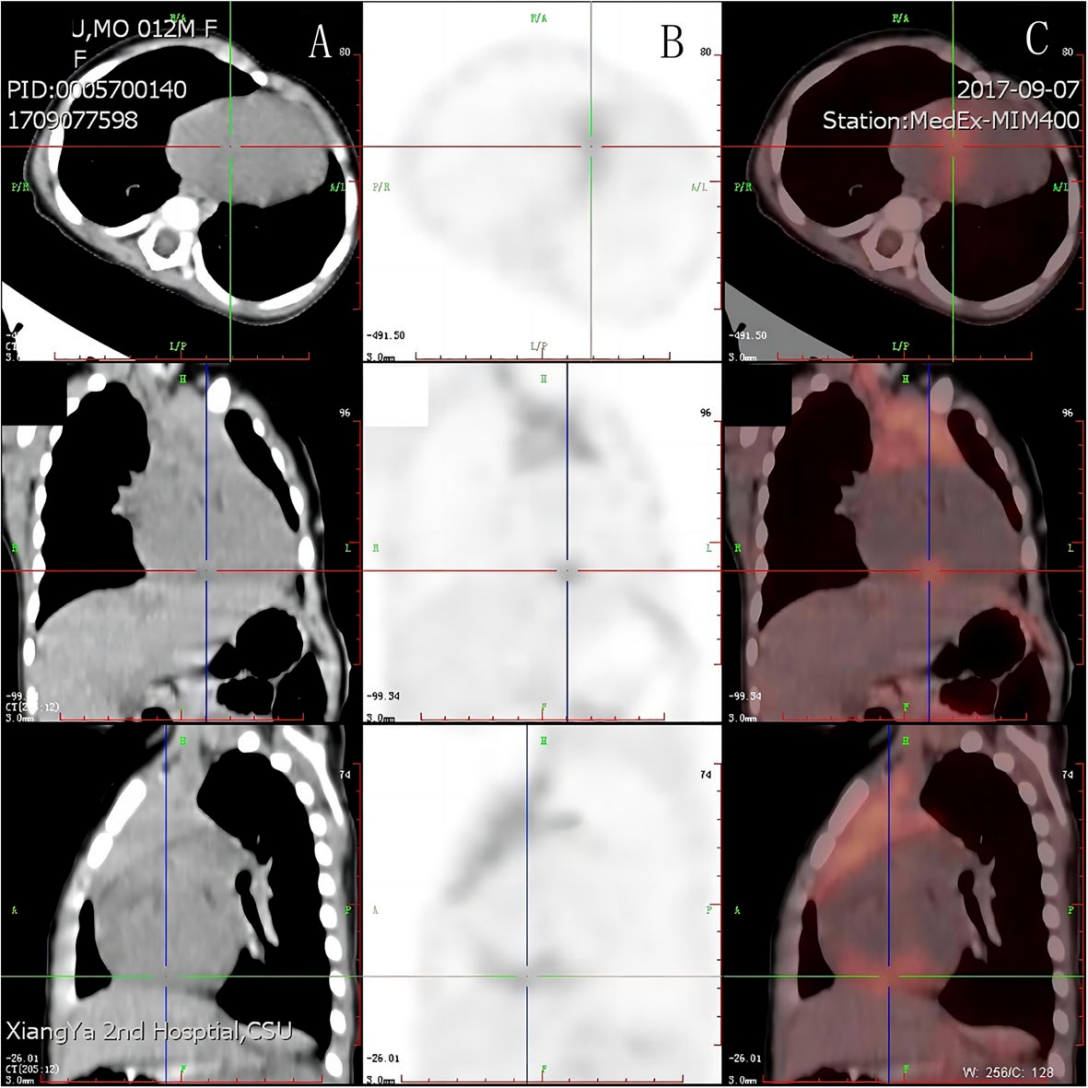
Computerized tomography scan (**A**) and positron emission tomography (**B, C**) confirmed the presence of the mass in the same area (cross area)

A 10-month-old female infant with history of pneumonia was diagnosed with a large atrial septal defect (ASD) 22*21 mm in size. The patient had a large atrial septal defect, a significant cardiac murmur, and recurrent pulmonary infections; additionally, the infant’s growth was significantly delayed and feeding was slightly difficult. Therefore, we decided to treat the child surgically because of the septal defect and the associated symptoms. When she underwent a routine pre-operation transthoracic echocardiography, it revealed an abnormal occupying lesion with high echogenicity 35*12*9 mm in size, stretching across from the anterior atrioventricular junction to the posterior atrioventricular junction at the bottom of the ventricular septal area. Transthoracic echocardiography revealed no hemodynamic changes due to the mass (Fig. 1A-C). A computerized tomography scan and positron emission tomography confirmed the presence of the mass in the same area (Fig. 2). The electrocardiogram showed sinus rhythm with incomplete right bundle branch block. Routine laboratory tests were ordered, and no abnormal results were found. No family history was obtained.

We observed two keys of dealing with this patient. The first was that ASD should be surgically repaired because the size of the orifice was too large to close by a device alone. The second was the strategy to deal with the trickiest part-the occupying lesion. The mass was impossible to resect and only biopsy with the occupying lesion was possible because of the position and the type of mass. The histopathological examination reported a benign tumor as hemangioma, and the patient was discharged without any symptoms on the fourth day after surgery and the tumor was given observation therapy. In the two-year follow-up period, the patient remained asymptomatic, and the size of the tumor remained unchanged (Fig. 1D,E).

## Discussion

Although ASD is the simplest congenital heart disease to repair, the optimal treatment timing for asymptomatic ASD is still unclear. However, most cardiologists agree that ASD with significant symptoms should be closed once the diagnosis is confirmed. In this case, transcatheter closure may not be feasible due to the large defect and early age.

**Fig. 3:**
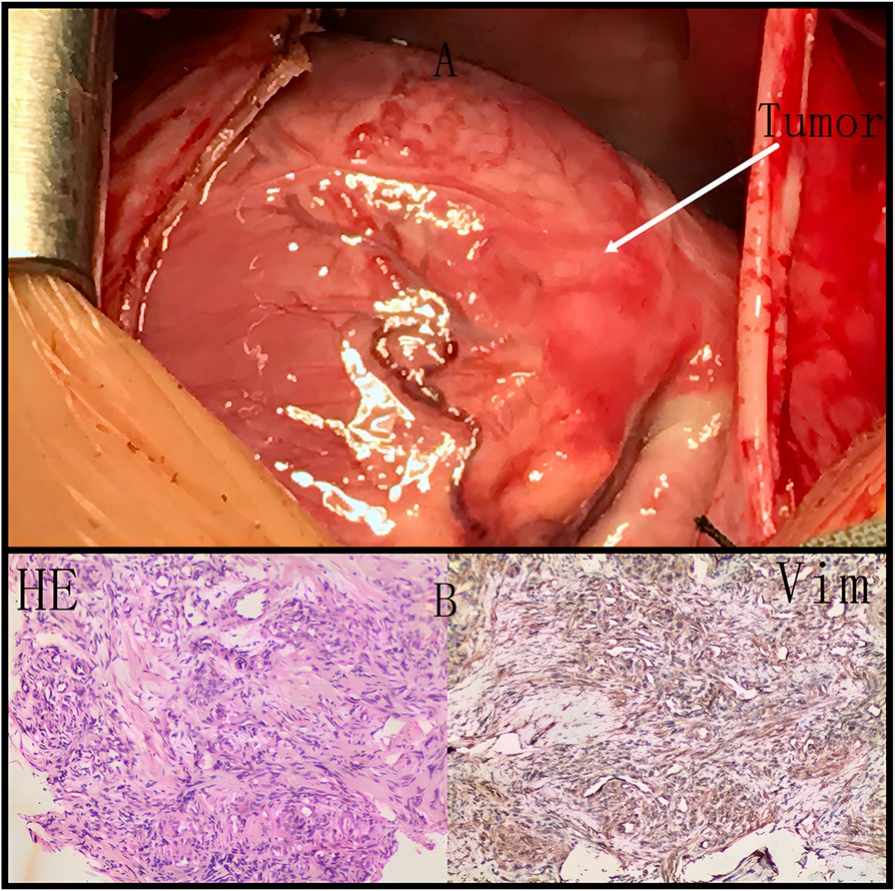
**A** Thoracotomy revealed a mass stretching across from the right atrial surface to the right ventricular surface; the mass had an irregular shape and a rubbery consistency. **B** The histopathological examination indicated a benign tumor as hemangioma (hematoxylin-eosin staining)

The incidence of cardiac tumor is 0.02%, and 75% of them are benign. The most common heart tumor in infants is rhabdomyoma [1], which was the first thing we considered in this case. We changed our view when we opened the patient’s chest and saw the tumor (Fig. 3A). The tumor, which stretched across from the right atrial surface to the right ventricular surface, had an irregular shape and a rubbery consistency. This tumor could be touched from the anterior atrioventricular junction to the posterior atrioventricular junction along the bottom of the heart. We reconfirmed that this was an unresectable tumor and performed a biopsy.

The coexistence of cardiac tumors and malformations of the heart may confuse physicians regarding the origin of the symptoms [2]. Some reports suggest that a space-occupying lesion may be associated with, or possibly induce, a malformation within the developing heart in some circumstances [2]. In this case, it was important to determine the histological characteristics of the tumor which may involve changes in hemodynamics or electrocardiogram results in the future. If a simple cardiac tumor is present, the ancillary tests become even more important. In this case, transthoracic echocardiograms and cardiac computerized tomography angiography(CTA) were done first after the child was admitted to the hospital. Transthoracic echocardiograms were the first to detect the cardiac mass, while the cardiac CTA did not indicate the cardiac mass, suggesting that cardiac CTA is not sensitive to hemangiomas. An MRI was not performed in this case, and we believe that cardiac MRI may have greater advantages in evaluating cardiac function and myocardial lesions. The most important test in this case was PET-CT, which determines the nature of the mass, its benignity and malignancy, and the presence of metastases.

The histopathological examination indicated a benign tumor, specifically a hemangioma (Fig. 3B). Hemangiomas in the heart are heterogenous with only a few present at birth or in infancy [3]. Only very few primary tumors and cysts of the pericardium, which are rare, are cardiac hemangiomas [4]. In children, the most common site of hemangioma is the right atrium, which may cause hydrops and cardiac tamponade because of large pericardial effusions [1]. Other common symptoms of hemangioma are atrial obstruction and changes in the electrocardiogram. In this case, no pericardial effusions were detected in surgery and no obstruction by the tumor was observed in echocardiography and no particular changes were observed in the electrocardiogram or electrocardiogram examination.

All symptomatic and hemodynamically compromised patients require surgical treatment [5]. Complete resection or even partial resection of benign cardiac tumors can provide strong early and long-term results. Nevertheless, only cardiac transplant should be considered for unresectable cardiac masses like this case if they seriously impair cardiac function [5]. In this case, we believe there was no impairment of cardiac function and therefore no need for resection of this tumor.

## Conclusion

Cardiac epicardium hemangiomas is a rare solid tumor of the heart. If the mass is impossible to resect and does not cause hemodynamic impairment, only mass biopsy is possible.

## Data Availability

The raw data supporting the conclusions of this article will be made available by the corresponding authors, without undue reservation.

## Abbreviations

ASD: Atrial septal defect
CTA: Computerized tomography angiography
